# Supplementary material for: Solution-state methyl NMR spectroscopy of large non-deuterated proteins enabled by deep neural networks
Source: Nat Commun. 2024 Jun 13;15:5073. doi: 10.1038/s41467-024-49378-8 (PMC11176362; doi:10.1038/s41467-024-49378-8)
Supplement: Supplementary file 1 — Supplementary Information [file 41467_2024_49378_MOESM1_ESM.pdf]

Supplementary Information

Solution-State NMR Spectroscopy of Large Non-Deuterated Proteins Enabled by Deep Neural Networks

Gogulan Karunanithy, Vaibhav Kumar Shukla, and D Flemming Hansen

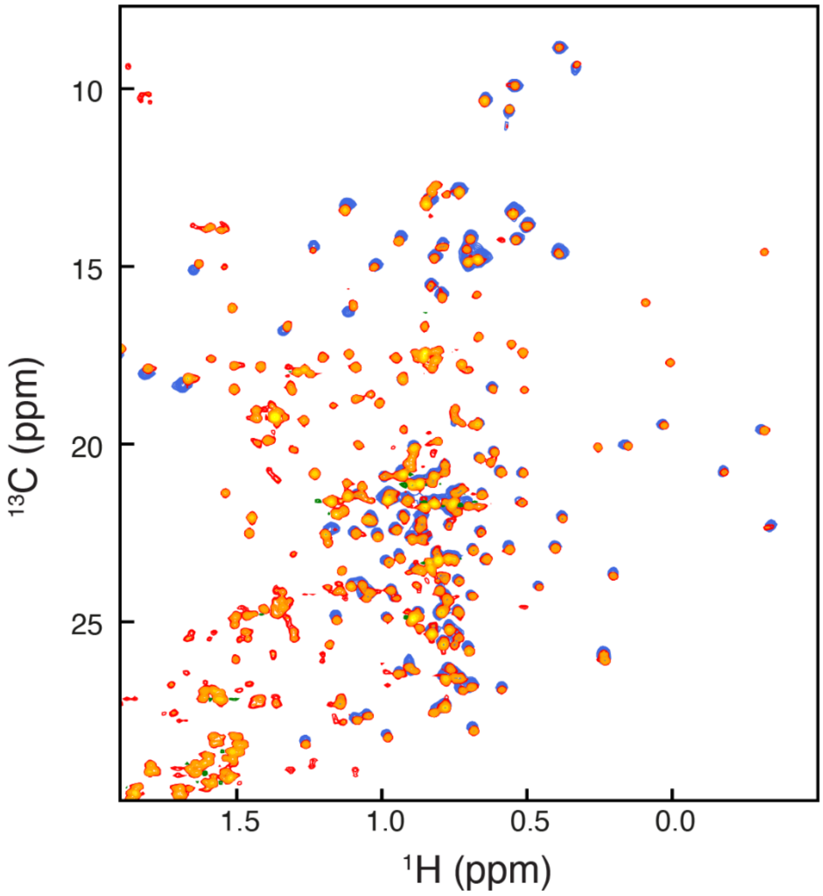

Supplementary Figure 1 | Comparison of FID-Net and classically processed spectra for HDAC8. Shown is a FID-Net processed HSQC spectrum in (orange) of the 42 kDa HDAC8 (Figure 3b) with a methyl-TROSY HMQC spectrum of an ILVM specifically labelled and deuterated HDAC8 (blue). Small shifts are due to two-bond (^{13}C) and three-bond (^{1}H) deuterium isotope shifts that are different for Isoleucine (2 gamma protons), Leucine (1 gamma proton), Valine (1 beta proton), and Methionine (0 delta protons).

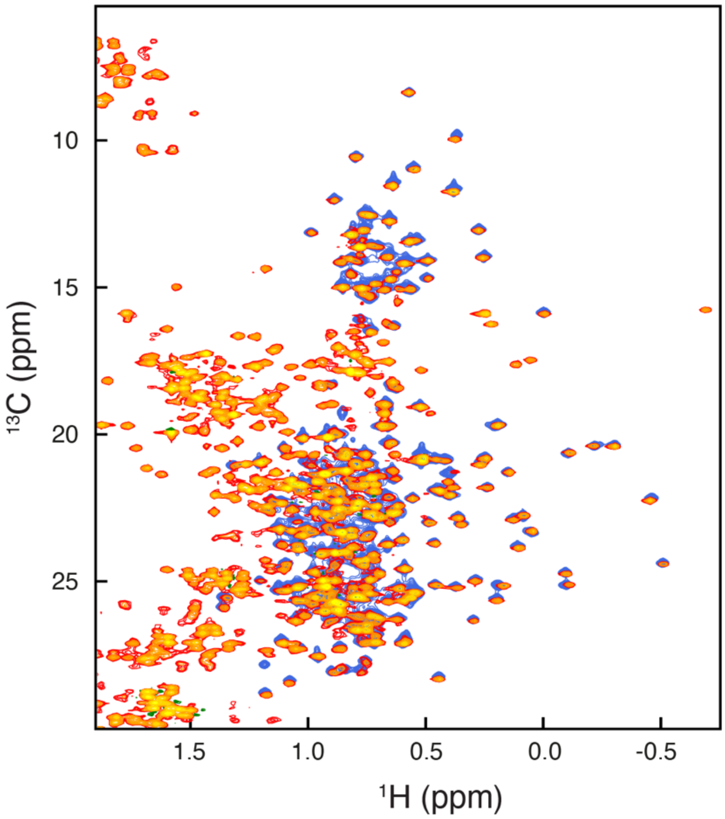

Supplementary Figure 2 | Comparison of FID-Net and classically processed spectra for MSG. Shown is a comparison of FID-Net processed HSQC spectrum in (orange) of the 80 kDa MSG (Figure 3e) with a methyl-TROSY HMQC spectrum of an ILV specifically labelled and deuterated MSG (blue). Small shifts are due to two-bond (^{13}C) and three-bond (^{1}H) deuterium isotope shifts that are different for Isoleucine (2 gamma protons) and Leucine (1 gamma proton) and Valine (1 beta proton).

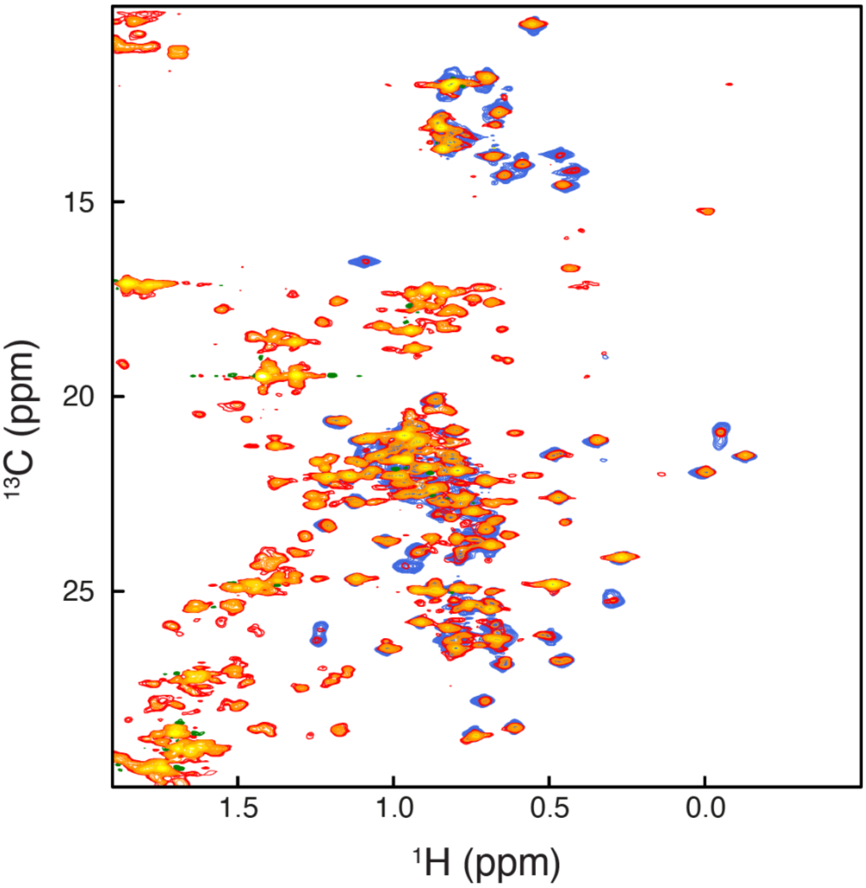

Supplementary Figure 3 | Comparison of FID-Net and classically processed spectra for α7α7. Comparison of FID-Net processed HSQC spectrum in (orange) of the 360 kDa α7α7 (Figure 4b) with a methyl-TROSY HMQC spectrum of an ILV specifically labelled and deuterated α7α7 (blue). Small shifts are due to two-bond (^{13}C) and three-bond (^{1}H) deuterium isotope shifts that are different for Isoleucine (2 gamma protons) and Leucine (1 gamma proton) and Valine (1 beta proton).

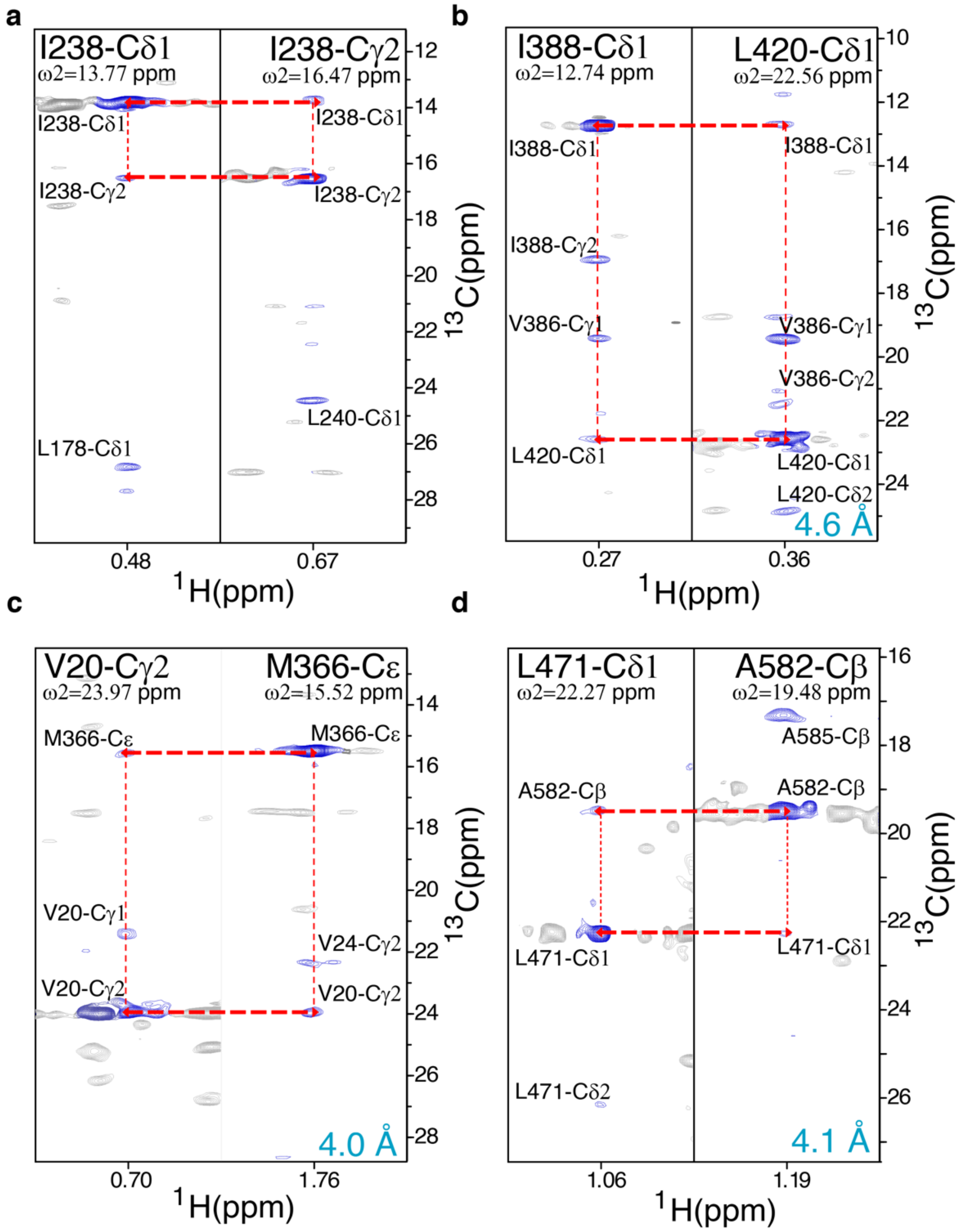

Supplementary Figure 4 | NOESY spectra of uniformly ^{13}C labelled, non-deuterated 80 kDa MSG. **(a)**-to-**(d)** 2D planes of the 3D ^{13}C-^{13}C-^{1}H NOESY spectra for methyl planes of **(a)** I238-$^{13}C^{\delta1}$ and I238-$^{13}C^{\gamma2}$. **(b)** I388-$^{13}C^{\delta1}$ and L420-$^{13}C^{\delta1}$. **(c)** V20-$^{13}C^{\gamma2}$ and M366-$^{13}C^{\varepsilon}$. **(d)** L471-$^{13}C^{\delta1}$ and A582-$^{13}C^{\beta}$.

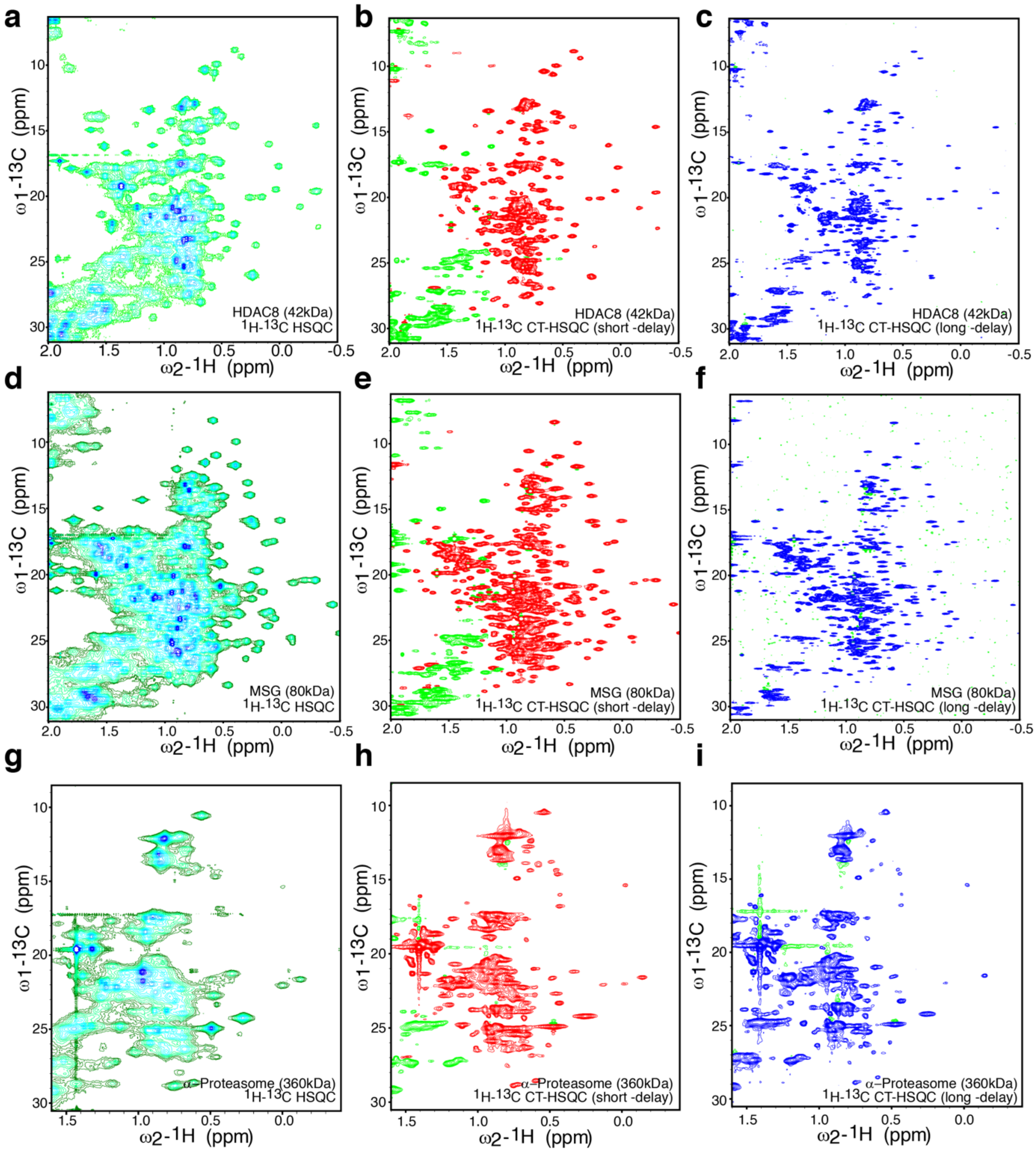

Supplementary Figure 5 | Comparison of ^{13}C HSQC and ^{13}C-Constant Time (CT)-HSQC spectra of uniformly ^{13}C labelled, non-deuterated HDAC8, MSG, and α7α7-proteasome. (**a**), (**d**), and (**g**) are the ^{13}C-HSQC spectra of HDAC8, MSG, and α7α7-proteasome, respectively. (**b**), (**e**), and (**h**) are the ^{13}C-constant-time (CT) HSQC spectra, acquired with constant-time period of 27 ms, of HDAC8, MSG, and α7α7-proteasome, respectively. (**c**), (**f**), and (**i**) are the ^{13}C-CT-HSQC spectra, acquired with constant-time of 54 ms, of HDAC8, MSG, and α7α7-proteasome, respectively.

Supplementary Table 1 | Parameter ranges used to train the ^{13}C decoupling and sharpening network.

Number of signals	10 – 150
Amplitude*	0 – 2.0
^{1}H dimension complex points	128 – 256
^{13}C dimension complex points	200 – 512
^{1}H dimension SW (Hz)	1500 – 5400
^{13}C dimension SW (Hz)	2500 – 8000
$^{1}J_{CC}$ (Hz)	28 – 40**
$R_2^{(1)}$ (s^{-1}) (^{1}H dimension)	5 – 150
$R_2^{(2)}$ (s^{-1}) (^{13}C dimension)	5 – 150

*Normal distribution with mean 1.0 and standard deviation of 0.5 that is truncated to between 0.0 - 2.0
**During training 5.0% of the $^{1}J_{CC}$ couplings are on average set to 0 Hz to mimic singlets.

Supplementary Table 2 | Parameter ranges used to train the ^{1}H sharpening network.

Number of signals	10 – 200
Amplitude*	0 – 2.0
1H dimension complex points	100 – 256
13C dimension complex points	200 – 512
1H dimension SW (Hz)	1500 – 5000
13C dimension SW (Hz)	2500 – 8000
^{1}J (Hz)	0.0
$R_2^{(1)}$ (s-1) (1H dimension)	5 – 150
$R_2^{(2)}$ (s-1) (13C dimension)	3 – 30

*Normal distribution with mean 1.0 and standard deviation of 0.5 that is truncated to between 0.0-2.0
